# Ellagic Acid Suppresses the Oxidative Stress Induced by Dietary-Oxidized Tallow

**DOI:** 10.1155/2018/7408370

**Published:** 2018-11-18

**Authors:** Alam Zeb, Adnan Akbar

**Affiliations:** ^1^Laboratory of Functional Foods Chemistry, Institute of Biochemistry, Technical University of Graz, Graz, Styria, Austria; ^2^Laboratory of Biochemistry, Department of Biotechnology, Faculty of Biological Sciences, University of Malakand, Chakdara, Khyber Pakhtunkhwa, Pakistan

## Abstract

Dietary tallow was thermally oxidized at 180°C in an open fryer. The oxidized tallow (OT) and unoxidized tallow were characterized for oxidation parameters and fatty acid composition using GC-MS. Tallow samples were fed to rabbits along with 50, 100, and 150 mg/kg/day of ellagic acid (EA) for three weeks. Results revealed that the peroxide value (PV) and thiobarbituric acid reactive substances (TBARS) significantly increased, while radical scavenging activity (RSA) of the tallow decreased significantly with oxidation. GC-MS analysis showed eight fatty acids in the tallow samples, where palmitic acid (48.5-49.7 g/100 g), linoleic acid (18.7-23.7 g/100 g), stearic acid (13.5-15.6 g/100 g), and margaric acid (6.32-6.42 g/100 g) were the major fatty acids. Animal studies showed that oxidized tallow (OT) alone or in combination with EA significantly altered the body weight of the rabbits. Serum biochemical parameters and renal function tests were affected by OT and ameliorated by EA. The toxic effects of OT on haematological indices were minimized by EA. The supplementation of OT alone had significant effects on the liver structure and functions. The coadministration of EA reduced the toxic properties of OT on the liver, by increasing the antioxidant (GSH) system. The rabbit heart was also affected by the OT, which was ameliorated by EA supplementation. These results suggested that the supplementation of EA was beneficial against the OT-induced oxidative stress and may be considered for foods containing oxidized lipids.

## 1. Introduction

Foods are prepared in dietary lipids such as fats or oils. During frying or food preparation, triacylglycerols are oxidized to form primary oxidation products. These primary oxidation products may include hydroperoxides, epoxides, hydroxides, and epoxides [1]. The lipid oxidation products formed are highly reactive. Most of these products are either free radicals or highly oxygenated compounds with high affinity for further reactions [2]. During the frying of foods, these products enter the food matrix and consequently consumed by humans [3]. The oxidized lipids then enter the gastrointestinal system, where it is metabolized or reaches the intestine as such. During digestion, these oxidized products alter several biochemical reactions thus causing toxicity [4]. The toxicity of the oxidized lipids may also be due to their capacity to form a complex with proteins. The oxidized products are toxic and are capable of inhibiting enzymatic reactions and cellular respiration and also contribute to ageing. The dietary lipid oxidation induces oxidative stress and increases lipogenesis in animal models [5, 6]. The lipogenesis was more pronounced in the liver and also in adipose tissues.

Dietary oxidized frying oil had been found to upregulate both hepatic acyl-CoA oxidase enzyme and one of the important genes (cytochrome p450 4 a1) in rats [7]. The dietary oxidized oil also activates peroxisome proliferator-activating receptors thus altering lipid metabolism. These effects were independent of the nature of the animal used [8]. The oxidized dietary lipids were also found to alter the glucose metabolism [9]. The oxidized lipids induced glucose intolerance by the mediation of altering the insulin secretion. The oxidized tallow was found to increase significantly the levels of serum total cholesterol, triglycerides, liver function tests, and liver toxicity [3]. Similarly, oxidized Vanaspati ghee was also causing significant detrimental effects on serum biochemical parameters, haematological parameters, and liver histology [10]. Fat accumulation in the liver was highly favoured by the ingestion of thermally oxidized sunflower oil [5]. Sea buckthorn seed oil [11], tomato products [6], and dietary glycine and glutamic acid [12] were found beneficial in controlling the oxidative stress induced by dietary oxidized lipids. The continuous uses of fried fats or oils in our daily food preparation have therefore warranted to determine other possible alternative ways for protection against the oxidative stress produced by dietary oxidized lipids. Phenolic compounds also played a significant role in the prevention of oxidative stress in animal models [13–15] and in humans [16]. Ellagic acid was found to be a potentially strong antioxidant in different kinds of oxidative stresses [17]. These studies only focused on the chemical-induced oxidative stress, while lacking its role against the oxidative stress produced by dietary oxidized lipids. This study was therefore aimed at determining the role of ellagic acid as a widely known oxidation suppressor against the oxidative stress produced by dietary oxidized oils.

## 2. Materials and Methods

### 2.1. Sample Collection

Samples of the animal adipose tissues (buffalos) were collected from the local slaughterhouse in Mingora Swat. Tallow was prepared as per the method of Zheng and Hanna [18]. After sample preparation, tallow was stored in the refrigerator for further analysis.

### 2.2. Thermal Oxidation of Tallow

Unoxidized tallow was taken as the control. Tallow (1 kg) was thermally oxidized in the open fryer at a controlled temperature of 180°C for 10 hours continuously. The oxidized samples were stored in the refrigerator until feeding to animals or characterization.

### 2.3. Characterization of Tallow

Peroxide values (meq/kg) were determined using an AOCS official method no Cd 8b-90 [19]. Thiobarbituric acid reactive substances (TBARS) were determined as per the optimized developed method [20]. Briefly, samples (100 mg) were dissolved in 10 mL of the glacial acetic acid. The extract (0.5 mL) was mixed with 0.5 mL of the thiobarbituric acid solution and kept in boiling water bath for 60 min. The absorbance of the samples was calculated at 532 nm against the blank. The values of TBARS were calculated from the standard calibration curve and expressed as mmol/g. The DPPH radical scavenging activity (RSA) was determined with 0.1 mM diphenyl-1-picrylhydrazyl (DPPH) solution using a UV-visible spectrophotometer (Shimadzu Japan) at 515 nm against methanol blank. The RSA was calculated as % RSA.

Fatty acids in the tallow samples were determined using gas chromatography coupled to mass spectrometry (GC-MS). For this purpose, 20 mg of the oil sample was converted to its methyl esters using acidified methanol [11]. The GC-MS system (QP-2010 plus) of the Shimadzu Corporation (Japan) was used for the analysis of fatty acid methyl ester of tallow samples. The total ion chromatograms (TIC) were recorded in the range of *m*/*z* 85-400. The amount of fatty acids was quantified using the available authentic standards and also by comparison with fatty acids MS library, where standards were not available. The fatty acids were expressed as g/100 g of the samples.

### 2.4. Animal Feeding

Rabbits as experimental animals were used. Rabbits were acclimatized to the new environment for one week. The experiments were conducted as per the approved protocols of animal care and experimentation, which are in accordance with the Helsinki Declaration. The experiments were approved by the Advanced Studies and Research Board of the University of Malakand. Male rabbits were randomly divided into six groups with five animals in each group according to the following scheme:

Group 1: control without any treatment on a normal diet
Group 2: fed with 50 mg/kg body weight of ellagic acid and oxidized tallow (3 g/kg) represented as OT + 50EA
Group 3: fed with 100 mg/kg body weight of ellagic acid and oxidized tallow (3 g/kg) represented as OT + 100EA
Group 4: fed with 150 mg/kg body weight of ellagic acid and oxidized tallow (3 g/kg) represented as OT + 150EA
Group 5: fed with 100 mg/kg body weight of ellagic acid only represented as 100EA
Group 6: fed with oxidized tallow (3 g/kg) only represented as OT

The doses of ellagic acid in the present study were lower than the values of 200 mg/kg as reported in the literature [21]. The oral gavage feeding was continued for 7 days, while the 8th day was considered a break for the collection of blood samples from the jugular vein of the rabbit.

### 2.5. Weight Changes

The changes in the weight of the whole body, heart, and liver were determined using a digital balance with high accuracy.

### 2.6. Serum Biochemical Parameters

Serum biochemical parameters such as total cholesterol, total triglycerides, and glucose were determined using the available standard reagent kits of HUMAN (Germany).

### 2.7. Renal Function Tests

The serum was analyzed for renal function tests, i.e., serum creatinine, urea, and uric acid, using the standard reagent kits of HUMAN (Germany).

### 2.8. Haematological Studies

Blood (3 mL) in the EDTA tubes was processed for the determination of different haematological parameters using the automatic CELL-DYN 3200 of the Abbott Diagnostic Division, Canada. The haematological parameters such as haemoglobin concentration, platelet concentration, monocytes, lymphocytes, eosinophils, total leucocyte counts, basophils, platelets, and neutrophils were determined.

### 2.9. Liver Structure and Function

The liver was analyzed for histological studies. Briefly, a small section of the median lobe of the liver was dissected. The section was then fixed in formalin buffer (10%) for 14 h. The dissected section was then dehydrated with ethanol solutions and further embedded in paraffin. Liver sections of about 8-10 mm in thickness were cut using a microtome, then deparaffinized, rehydrated again with ethanol, and finally stained on the slide [12]. The final slides were then studied using a microscope with a 1.3 MP digital camera. The level of the liver inflammation marker was studied as serum alanine aminotransferase activity was measured using HUMAN (Germany) kits and expressed as U/L.

Lipids from the liver tissues were extracted using the optimized method [10]. The DPPH radical scavenging activity of the liver lipids was determined with 0.1 mM diphenyl-1-picrylhydrazyl (DPPH) solution freshly prepared in methanol. For this purpose, 1.95 mL of the DPPH solution was mixed with 0.05 mL of the sample extract. The mixture was incubated for 30 min in a dark chamber. The absorbance of the mixture was recorded using a UV-visible spectrophotometer (Shimadzu Japan) at *λ* 515 nm against methanol blank. The RSA was calculated as % RSA.

### 2.10. Total Reduced Glutathione

Reduced glutathione from the liver was extracted using 10 mL of ice-cold metaphosphoric acid (3%). A sample extract of 0.5 mL was mixed with 5 *μ*g of reduced glutathione, 1.5 mL of potassium sodium phosphate buffer (0.5 M, pH 8.0), and 0.03 mL of dithio nitrobenzene (DTNB). After 3 min of shaking, the absorbance of the sample was determined at 412 nm against the reference blank [22]. The amount of the total reduced glutathione was expressed as mmol/g.

### 2.11. Glutathione S-Transferase Activity

The liver was also analyzed for glutathione S-transferase (GST) activity. Glutathione transferase is a highly selective hydrolyzing enzyme for detoxification of glutathione conjugates with different stressing or carcinogenic compounds. GST activity was measured using a 1-choloro-2,4-dinitrobenzene (CDNB) reagent with a concentration of 1 mmol/L. The extract (100 *μ*L) prepared in ethanol and buffer was mixed with 100 *μ*L CDNB, 100 *μ*L of GSH, and 700 *μ*L of potassium phosphate buffer (pH 7.5) [23]. The enzyme activity was expressed as unit/g.

### 2.12. Histology of the Heart

A rabbit heart was analyzed for histological studies. Briefly, a small section of the heart was dissected. The section was then fixed in formalin buffer (10%) for 14 h. The dissected section was then dehydrated with ethanol solutions and further embedded in paraffin. The sections (8-10 mm in thickness) were cut using a microtome, deparaffinized, rehydrated again with ethanol, and finally stained on the slide [24]. The final slides were then studied using a microscope with a 1.3 MP digital camera.

### 2.13. Statistical Analysis

Each parameter was repeated three to five times and was expressed as the mean with standard deviation. The data were evaluated for the statistical significance using one-way analysis of variance with the post hoc test of Tukey's at *α* = 0.05 using GraphPad Prism 7.0 (GraphPad Software Inc., 2016).

## 3. Results and Discussion

### 3.1. Characterization of Tallow

Table 1 shows the peroxide values, TBARS, and radical scavenging activity of oxidized and control tallow. The peroxide value of the control tallow was 9.03 meq/kg and increased to 111.3 meq/kg upon oxidation. This showed that oxidation of tallow increased the PV of the tallow. The results are in agreement with findings [3, 6]. The increase in the PV may be due to the oxidation of triacylglycerols present in the tallow to form hydroperoxides, which are measured as PV values. Similarly, the TBARS value of the control tallow was 0.254 mmol/g and increased to 0.518 mmol/g on oxidation. However, the amount of TBARS was relatively very low as compared to the thermally oxidized edible oils under similar conditions of sunflower oil [25] and rapeseed oils [12]. The DPPH radical scavenging activity was 51.1% and decreased to 40.5% upon oxidation. The results are in agreement with previous findings [6]. There was a strong positive correlation coefficient (*R* ^2^ = 0.9999) between the increase in the PV and TBARS and a strong negative correlation (*R* ^2^ = 0.9999) between TBARS or PV and RSA values. The formation of peroxides and secondary oxidation compounds resulted in the reduction of antioxidant activity of the tallow.

Eight fatty acids were identified and quantified in the present work using total ion chromatogram (TIC) of GC-MS as shown in Table 1. It has been observed that tallow was rich in palmitic acid, i.e., 48.5 and 49.7 g/100 g, in control and oxidized tallow. Linoleic acid was 23.47 g/100 g and reduced to 18.7 g/100 g on oxidation. Stearic acid was the third most abundant fatty acid in the tallow, while margaric acid and myristic acid were placed on the fourth and fifth positions, respectively. The amount of palmitic acid was higher than that reported by Segura et al. [26], which was 25.7 g/100 g. The authors, however, reported a higher amount of stearic acid (26.7 g/100 g) than the present results. This might be due to the difference in the type of organism used for the extraction of tallow. The presence of a high amount of linoleic acid in tallow may be one of the contributing factors for specific taste and flavour of foods during frying.

### 3.2. Effects on Body Weight Change

Table 2 shows the comparison of the body weight change with respect to the control values of the first week. In control rabbits, the body weight increased to 26.3 g and 37.3 g in the third week. In the first week of the OT + 50EA group, there was a significant increase in the body weight, which was equal to the body weight in the third week of the control group. The values of the net weight change were reduced with treatment times in the second and third weeks, respectively. There was also a significant decrease in the whole body weight changes with supplementation of OT with 100 mg/kg of EA. The decrease in weight was recorded with respect to the time of treatment. Similarly, for OT with 150EA, body weight increased with treatment time. There was a significant decrease in the body weight by the supplementation of EA at a dose rate of 100 mg/kg and also in the case of OT. However, the EA showed significant weight loss as compared to the OT supplementation. These results suggested that ellagic acid significantly contributed to the weight loss in the rabbits. The antiglycative and antidiabetic properties of the ellagic acid [27] may be contributing to the body weight loss in rabbits.

### 3.3. Effects on Serum Biochemical Parameters

**Figure 1:**
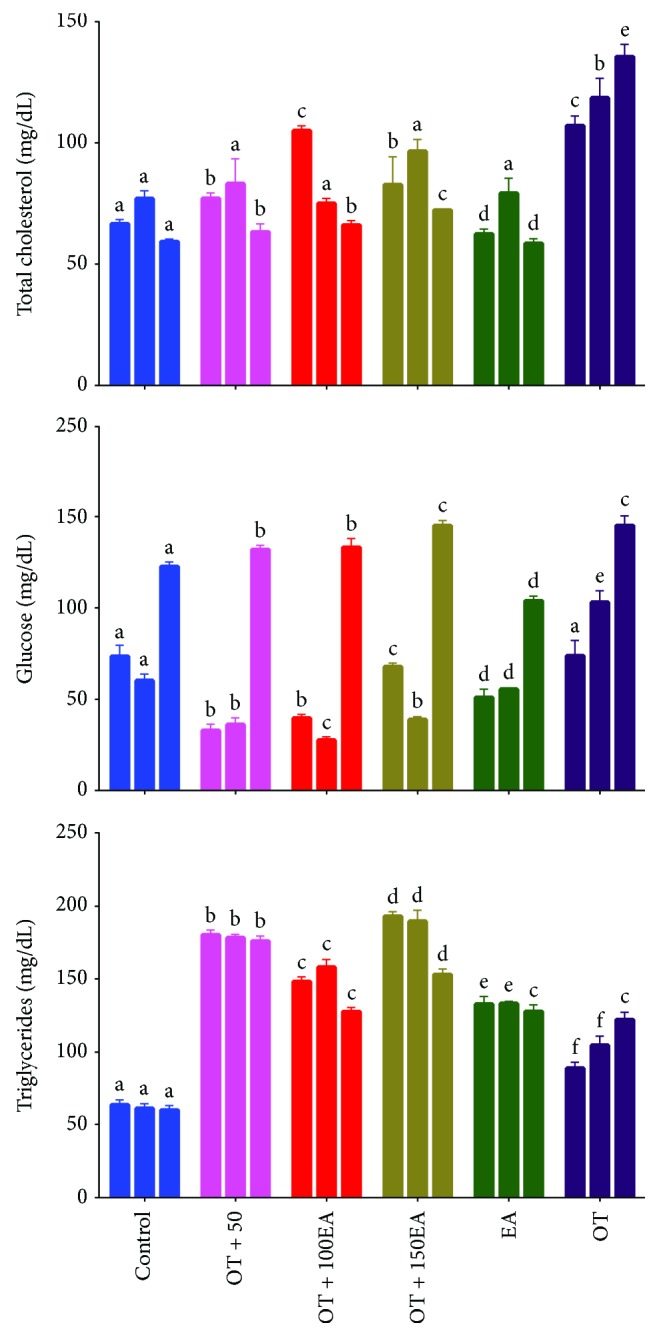
Effects of EA and OT alone or in combination on serum total cholesterol, glucose, and triglycerides. OT is oxidized tallow; EA is ellagic acid; 50, 100, and 150 are dose amounts in mg/kg. The three bars are weekly treatment starting from the left side. Different letters (a-f) in the same treatment week are a significant difference at *α* = 0.05 using Tukey's test.

Figure 1 shows the effects of ellagic acid on the serum total triglycerides, total cholesterol, and serum glucose levels of rabbits administered against the oxidized tallow. Ellagic acid was found to increase the serum triglycerides and total cholesterols upon coadministration with oxidized tallow. The results are in agreement with those of Klingenberg et al. [28], who showed that supplementation of tallow and high oleic sunflower oil increased the serum total triglycerides and cholesterols. These results also confirm the previous findings from our lab [6], where it was shown that thermally oxidized tallow increased the serum lipids in rabbits. In the present work, it was suggested that ellagic acid induced the synthesis of triglycerides in the experimental animals. The reduction in the serum glucose by the ellagic acid alone or in combination with EA for two weeks may be attributed to the antidiabetic potential of the ellagic acid recently reported [29]. It was suggested that ellagic acid stimulates insulin secretion by the *β*-cells of the pancreas and ultimately decreases glucose intolerance.

### 3.4. Effects on Renal Function

**Figure 2:**
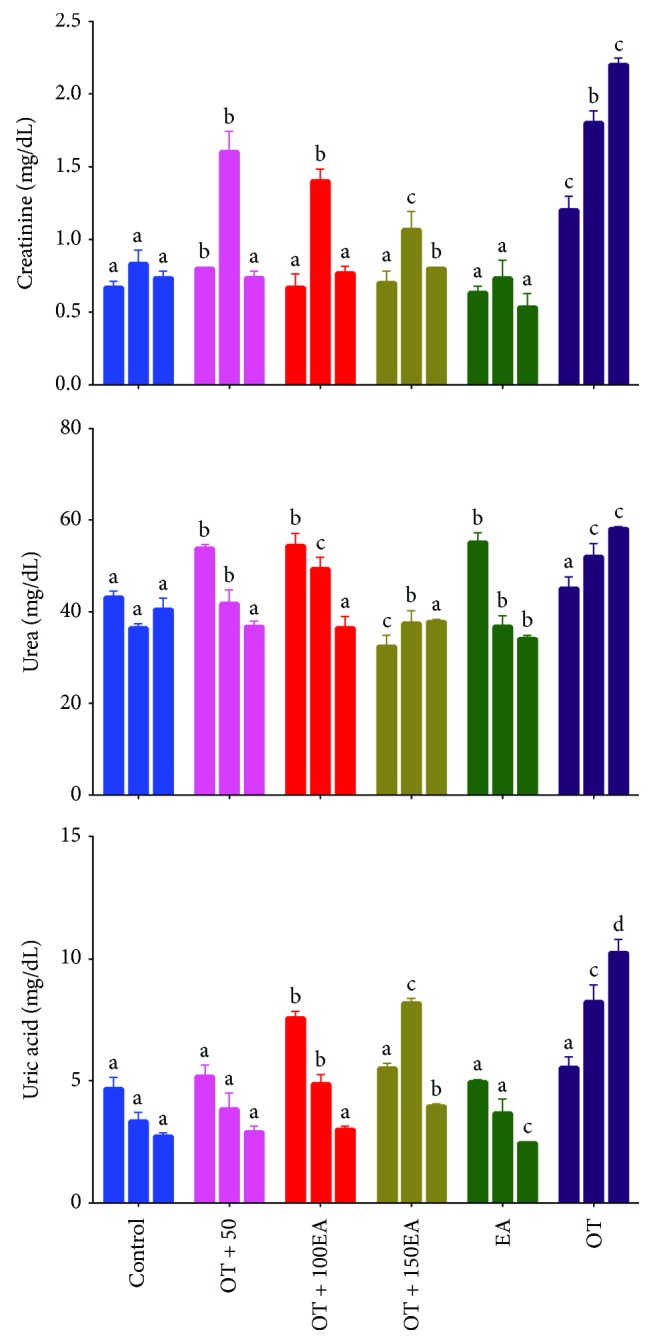
Effects of EA and OT alone or in combination on serum liver function tests. OT is oxidized tallow; EA is ellagic acid; 50, 100, and 150 are dose amounts in mg/kg. The three bars are weekly treatment starting from the left side. Different letters (a-d) in the same treatment week are a significant difference at *α* = 0.05 using Tukey's test.

Figure 2 shows the effects of ellagic acid on the serum renal function tests of rabbits administered against the oxidized tallow. It was observed that OT increased the serum creatinine, a marker of renal function, and was significantly reduced by ellagic acid. Similarly, serum urea and uric acid were also controlled by the cosupplementation of OT and EA in a dose-dependent manner. Atessahin et al. [30] showed that EA significantly reduced high plasma creatinine, urea, and calcium and also neutralized the harmful effects of cisplatin on oxidative stress markers. These authors also showed that EA improved cisplatin-induced pathological changes, which include tubular necrosis, degeneration, and tubular dilatation. They concluded that EA has a protective effect against cisplatin-induced oxidative stress and nephrotoxicity in rats, but the effects were not sufficient to prevent cisplatin-induced renal dysfunction. Similarly, Ahad et al. [31] showed that ellagic acid was protective for kidneys in diabetic rats partly through antihyperglycemia, which was believed to be accompanied by a reduction of inflammatory processes through inhibition of the NF-*κ*B pathway system. Thus, it was concluded that EA is beneficial in renal function by controlling the oxidative stress induced by thermally oxidized lipids.

### 3.5. Effects on Haematological Indices

It has been observed that white blood cells (WBC) increased significantly with EA alone or in combination with OT and EA (Table 3). A significant decline was also observed in lymphocytes (LYM) in the OT + 50EA group, while no significant changes in other treated groups. Granulocytes (GRAN) and red blood cells (RBC) were not affected by any treatment. Haemoglobin was found to decrease only in the OT + 150EA group, while no significant changes were observed in other groups. Similarly, a significant decline was also observed in EA alone or in combination with 150EA in haematocrit (HCT) values. There was no significant difference in the amounts of MCV and MCH, respectively. Platelets were significantly lower in all treated groups as compared to the control. Zeb and Rahman [3] and Zeb and Haq [6] showed that oxidized tallow significantly affected different haematological parameters. Thus, remedies are needed such as tomato powder and sea buckhorn oil, while this study provides the beneficial properties of an ellagic acid. Chao et al. [32] showed that ellagic acid has anticoagulation properties in mice. This means that EA is beneficial for haematological parameters and ultimately the heart. Similarly, Ikewuchi et al. [33] reported that the plant consists of large amounts of ellagic acid ameliorates, haematological indices, and oxidative stress produced in alloxan-induced rats.

### 3.6. Effects on Liver Structure and Function

**Figure 3:**
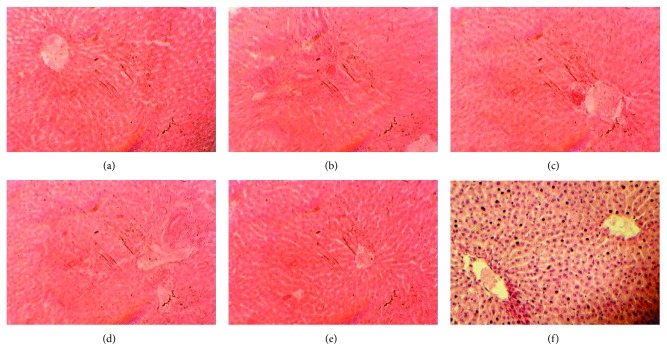
Effects of ellagic acid on the liver structure and function: (a) control, (b) OT + 50EA, (c) OT + 100EA, (d) OT + 150EA, (e) EA, and (f) OT. The pictures were documented at a magnification of 40x.

**Figure 4:**
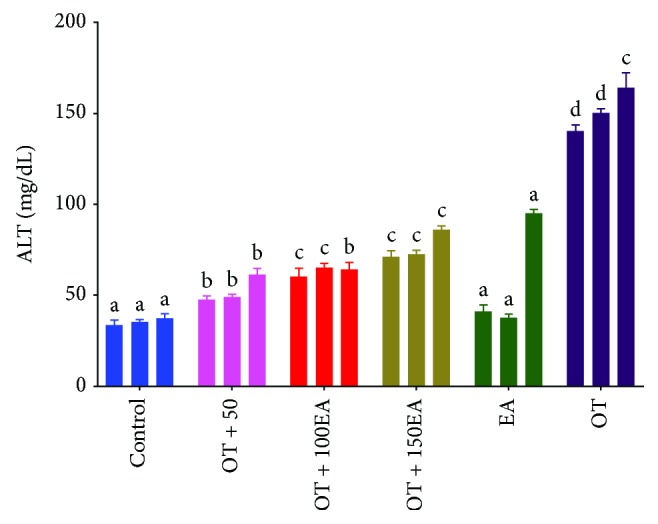
Effects of EA and OT alone or in combination on serum ALT levels. OT is oxidized tallow; EA is ellagic acid; 50, 100, and 150 are dose amounts in mg/kg. The three bars are weekly treatment starting from the left side. Different letters (a-d) in the same treatment week are a significant difference at *α* = 0.05 using Tukey's test.

The liver of the control rabbits was of normal structure and function. The supplementation of OT induced inflammation and toxicity in the rabbit's liver (Figures 3(a)–3(f)). The OT also elevates serum ALT levels, which was controlled by the EA supplementation (Figure 4). The low dose of EA, i.e., 50 mg/kg, was more effective than other higher doses. Liver RSA was increased by the supplementation of EA (Table 4). It was also found that EA normalizes the negative effects and oxidative stress induced by dietary oxidized tallow on the function and architecture of the liver. Singh et al. [34] showed that ellagic acid provides protection against the carbon tetrachloride-induced hepatotoxicity in rats. Ellagic acid was also found to be protective against the toxicity produced by paracetamol in rats [35] and was much better in CCl_4_-induced toxicity [36]. The present study was also in accordance with the study reported by Kannan and Quine [14], who showed that EA significantly ameliorates the toxic properties of isoproterenol in rats. The authors also reported that EA increased the antioxidant enzyme system of the liver.

### 3.7. Effects on Reduced Glutathione

**Figure 5:**
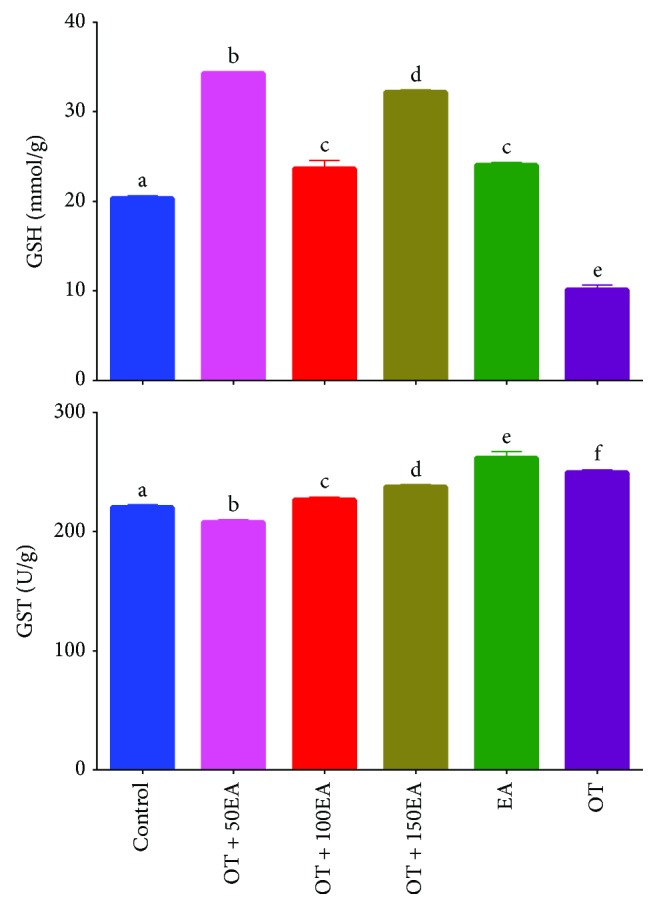
Effects of EA and OT alone or in combination on serum GSH & GST levels. OT is oxidized tallow; EA is ellagic acid; 50, 100, and 150 are dose amounts in mg/kg. Different letters (a-f) in the same parameter are a significant difference at *α* = 0.05 using Tukey's test.

Reduced glutathione (GSH) level is highly important for studying the oxidative stress on the biological system. Glutathione is a tripeptide with strong biological antioxidant properties. The effects of ellagic acid and oxidized tallow on the reduced glutathione (GSH) levels of the rabbit's liver have been presented. The amount of GSH was 20.3 mmol/g and significantly reduced to 10.2 mmol/g in an OT-fed rabbit's liver (Figure 5). The amount of GSH in the EA-fed group was close to the values of OT with 100EA. Similarly, the amounts of GSH in the OT + 50EA and the OT + 150EA groups were similar. Arafat et al. [37] showed that EA protected the liver and increased the amount of GSH in the liver. Similarly, El-Shitany et al. [38] also showed that EA enhanced the production of GSH and consequently the oxidative stress. These results showed that oxidative stress produced by the dietary oxidized lipids can also be counteracted by ellagic acid.

### 3.8. Glutathione S-Transferase Activity

Glutathione S-transferase (GST) is a highly selective group of enzymes, which are required for the detoxification of foreign chemicals such as dietary oxidized lipids. The liver is the main site for the activity of this enzyme. Figure 5 shows the effects of ellagic acid and thermally oxidized tallow on the GST activity of the rabbit's liver. It was observed that the control group has a GST activity of 220.7 U/g of the tissue, which was significantly increased with the supplementation of OT. The activity was also found to rise by EA alone, which was higher than the OT group. There were no significant changes in the activity levels in the OT with the EA groups except in the OT + 50EA group, where the amount was lower than control levels. Previous results showed that EA enhanced the production of GST of the liver in nickel-induced oxidative stressed rats [39]. Pari and Sivasankari [40] also reported the increase in the GST of the liver, which was depleted by cyclosporine A in rats. Thus, an increase in the GST activity of the liver was due to the supplementation of EA to rabbits.

### 3.9. Effects on Histology of the Heart

**Figure 6:**
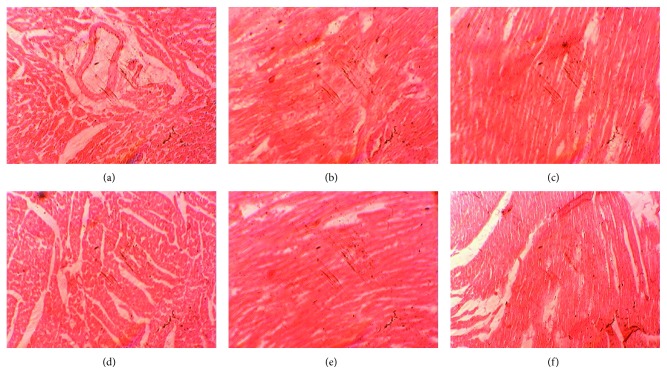
Effects of ellagic acid and oxidized tallow alone or in combination on the histology of the rabbit heart: (a) control, (b) OT + 50EA, (c) OT + 100EA, (d) OT + 150EA, (e) EA, and (f) OT. The pictures were documented at a magnification of 40x.

In control rabbits, there was a normal heart structure and function, while the OT + 50EA group revealed mild toxicity or myocarditis. No significant changes were observed in the OT + 100EA group, while the OT + 150EA group showed mild toxicity or myocarditis (Figure 6). This showed that lower doses of EA were protective for the heart against the OT-induced toxicity. Larrosa et al. [41] reviewed that ellagic acid is beneficial for vascular health. Similarly, ellagic acid was found to inhibit the myocardial necrosis and infarction induced by isoproterenol in rats [14] by protecting the mitochondria of the heart cells [42, 43]. It was also found that dietary ellagic acid provides protection against oxidant-induced endothelial dysfunction and atherosclerosis partly via Nrf2 activation [44]. Thus, it was suggested that EA was protective in the dose range of 50-100 mg/kg for the heart against the oxidative stress in the cardiovascular system produced by dietary oxidized tallow in rabbits.

## 4. Conclusions

Results revealed that PV and TBARS increased, while RSA of the tallow decreased significantly with oxidation at 180°C in an open fryer. A total of eight fatty acids were quantified in the tallow; palmitic acid, stearic acid, linoleic acid, margaric acid, and myristic acid were the major fatty acids. Animal studies showed that OT alone or in combination with EA significantly alters the body weight of the rabbits. Serum biochemical parameters and renal function tests were affected by OT and ameliorated by EA. The toxic effects of OT on haematological indices were minimized by EA. The supplementation of OT alone had significant effects on the liver structure and functions. The coadministration of EA reduced the toxic properties of OT on the liver, by increasing the antioxidant (GSH) system. The rabbit heart was also affected by the OT, while no significant effects were observed by the EA supplementation up to a dose rate of 100 mg/kg, suggesting an ameliorative effect at low doses of EA. These results suggested that the supplementation of EA was beneficial against the OT-induced oxidative stress and may be considered for foods containing oxidized lipids. Studies are therefore required to explore the sources of ellagic acid in different unexplored plants and its uses in food industries.

## Figures and Tables

**Table 1:** Quality characteristic and composition of fatty acids of control and oxidized tallow.

No.	Fatty acid/parameter	Code	Retention time (min)	Composition (g/100 g)^∗^	
				Control	Oxidized
1	Myristic acid	C14:0	12.9	3.56 ± 0.1^a^	3.24 ± 0.2^a^
2	Pentadecanoic acid	C15:0	14.6	1.37 ± 0.03^a^	1.76 ± 0.05^b^
3	Palmitic acid	C16:0	16.2	48.5 ± 0.2^a^	49.7 ± 0.1^b^
4	Margaric acid	C17:0	18.0	6.42 ± 0.2^a^	6.32 ± 0.2^a^
5	Stearic acid	C18:0	19.2	13.5 ± 0.2^a^	15.6 ± 0.1^b^
6	Linoleic acid	C18:2	19.6	23.7 ± 0.3^a^	18.7 ± 0.1^b^
7	Eicosanoic acid	C20:1	22.8	1.39 ± 0.1^a^	1.19 ± 0.05^b^
8	Behenic acid	C22:0	26.9	0.45 ± 0.05^a^	ND^b^
*Quality characteristics*					
1	PV (meq/kg)^∗^			9.03 ± 0.5^a^	111.3 ± 3.0^b^
2	TBARS (mmol/g)^∗∗^			0.254 ± 0.01^a^	0.518 ± 0.01^b^
3	RSA (%)^∗∗∗^			51.1 ± 0.4^a^	40.5 ± 0.8^b^

The identification of each fatty acid was based on comparison with authentic standards and comparison with reported in the library. Data are the mean of replicate (*n* = 5) readings and represented as the mean with a standard deviation. Different letters (a-b) in the same parameter showed a significant difference at *α* = ^∗^0.05, ^∗∗^0.01, and ^∗∗∗^0.001 using Tukey's test. PV: peroxide values; TBARS: thiobarbituric acid reactive substances; RSA: radical scavenging activity; C: carbons in fatty acid; ND: not detected.

**Table 2:** Effects of ellagic acid on the net changes in the whole body weight of the rabbits.

Duration (weeks)	Net body weight change (g)					
	Control	OT + 50EA	OT + 100EA	OT + 150EA	100EA	OT
1	0.0^a^	36.3^b^	−418.7^c^	−443.3^d^	−245.2^e^	−48.5^f^
2	26.3^a^	22.3^a^	−431.0^b^	−471.3^c^	−266.3^d^	−62.3^e^
3	37.3^a^	5.66^b^	−438.4^c^	−511.7^d^	−312.6^e^	−84.3^f^

Data are the mean of replicate (*n* = 5) readings and represented as the mean. Different letters (a-f) in the same week represent the significant difference at *α* = 0.05 using Tukey's test. OT represents oxidized tallow; EA is ellagic acid; 50, 100, and 150 represent the respective dose amounts in mg per kg of rabbit body weight.

**Table 3:** Effects of ellagic acid and oxidized tallow alone or in combination on the haematological parameters of rabbits.

Parameter	Control			OT + 50EA			OT + 100EA			OT + 150EA			100EA			OT		
	1	2	3	1	2	3	1	2	3	1	2	3	1	2	3	1	2	3
WBC (cells/*μ*L) × 10^6^	5.53^a^	7.23^b^	7.16^b^	6.93^b^	6.03^a^	5.86^a^	6.7b	7.83^c^	6.76b	6.1^a^	5.86^a^	6.16^a^	9.23^d^	7.16^b^	8.03^f^	6.0^a^	5.3^a^	5.2^a^
LYM (%)	43.0^a^	35.0^b^	32.0^c^	30.9^c^	31.1^c^	30.8^c^	44.9a	40.9^a^	39.2a	39.1^a^	39.0^a^	39.0^a^	30.8^a^	27.7^d^	33.3^c^	78.5^e^	79.0^e^	77.5^e^
RBC (cells/*μ*L) × 10^6^	5.12^a^	5.40^a^	5.09^a^	4.9^b^	5.34^a^	4.91^b^	3.90c	4.53^c^	4.13c	5.13^a^	4.36^b^	4.79^b^	5.01^b^	4.97^b^	5.69^a^	5.27^a^	5.94^a^	5.39^a^
HGB (g/dL)	11.4^a^	12.0^a^	12.0^a^	11.5^a^	11.9^a^	12.4^a^	9.96b	12.2^a^	12.1a	11.3^a^	9.9^b^	8.73^b^	10.7^a^	10.7^a^	10.7^a^	11.2^a^	12.4^a^	12.2^a^
HCT (%)	40.0^a^	40.6^a^	41.0^a^	36.8^b^	39.0^a^	39.7^a^	41.5a	41.5^a^	40.7a	35.2^b^	33.5^b^	31.4^b^	33.0^b^	33.4^b^	31.8^b^	39.2^a^	28.5^c^	30.2^b^
MCV (fL/cell)	65.5^a^	68.4^a^	68.9^a^	68.1^a^	68.8^a^	67.3^a^	66.6a	63.9^b^	64.5a	61.0^b^	64.3^a^	61.8^b^	59.5^b^	60.0^b^	60.4^b^	63.3^b^	64.0^b^	63.4^b^
MCH (pg/cell)	22.4^a^	19.7^b^	22.7^a^	24.8^a^	25.8^c^	22.4^a^	23.0a	23.8^a^	21.6a	26.6^c^	20.4^b^	18.2^b^	19.5^b^	20.0^b^	20.5^b^	18.2^b^	19.7^b^	19.2^b^
PLT (cells/*μ*L) × 10^3^	3.57^a^	3.39^a^	2.58^b^	2.49^b^	2.75^b^	2.47^b^	2.06c	2.84^b^	3.07b	2.10^c^	1.75^c^	1.74^c^	2.17^c^	2.04^c^	2.14^c^	2.45^b^	2.47^b^	2.56^b^

Data are the mean of replicate (*n* = 3) readings and represented as the mean. Different superscript letters (a-e) in the same treatment week represent the significant difference at *α* = 0.01 using Tukey's test. EA is ellagic acid; OT is oxidized tallow; 50, 100, and 150 represent the respective dose amounts in mg per kg of rabbit body weight. WBC: white blood cells; LYM: lymphocytes; RBC: red blood cells; HGB: haemoglobin; HCT: haematocrit; MCV: mean corpuscular volume; MCH: mean corpuscular haemoglobin; PLT: platelet count.

**Table 4:** Effects of ellagic acid supplementation against the oxidative stress induced by thermally oxidized tallow on the DPPH radical scavenging activity of the liver lipids.

Duration (weeks)	DPPH radical scavenging activity (%)					
	Control	OT + 50EA	OT + 100EA	OT + 150EA	100EA	OT
1	33.7 ± 2.6^a^	47.7 ± 2.0^b^	60.3 ± 4.4^c^	71.3 ± 3.0^d^	41.3 ± 3.3^e^	23.7 ± 2.1^f^
2	35.3 ± 1.2^a^	49.3 ± 1.2^b^	65.3 ± 2.0^c^	72.7 ± 2.0^d^	37.7 ± 2.0^a^	25.2 ± 1.2^e^
3	37.3 ± 2.4^a^	61.3 ± 3.2^b^	64.3 ± 3.6^b^	76.0 ± 2.1^c^	95.0 ± 2.1^d^	27.3 ± 2.0^e^

Data are the mean of replicate (*n* = 5) readings. Different superscript letters (a-f) in the same treatment week represent the significant difference at *α* = 0.05 using Tukey's test. DPPH represents diphenyl-1-picrylhydrazyl; OT represents oxidized tallow; EA is ellagic acid; 50, 100, and 150 represent the respective dose amounts in mg per kg of rabbit body weight.

## Data Availability

The data used to support the findings of this study are available from the corresponding author upon request.

## Supplementary Materials

Supplementary Materials Figure: representative total ion chromatogram (TIC) of fatty acids using GC-MS of tallow: (A) control tallow and (B) oxidized tallow.
