# Supplementary material for: Ellagic Acid Suppresses the Oxidative Stress Induced by Dietary-Oxidized Tallow
Source: Oxid Med Cell Longev. 2018 Nov 18;2018:7408370. doi: 10.1155/2018/7408370 (PMC6276410; doi:10.1155/2018/7408370)
Supplement: Supplementary Materials — Figure: representative total ion chromatogram (TIC) of fatty acids using GC-MS of tallow: (A) control tallow and (B) oxidized tallow. [file 7408370.f1.pdf]

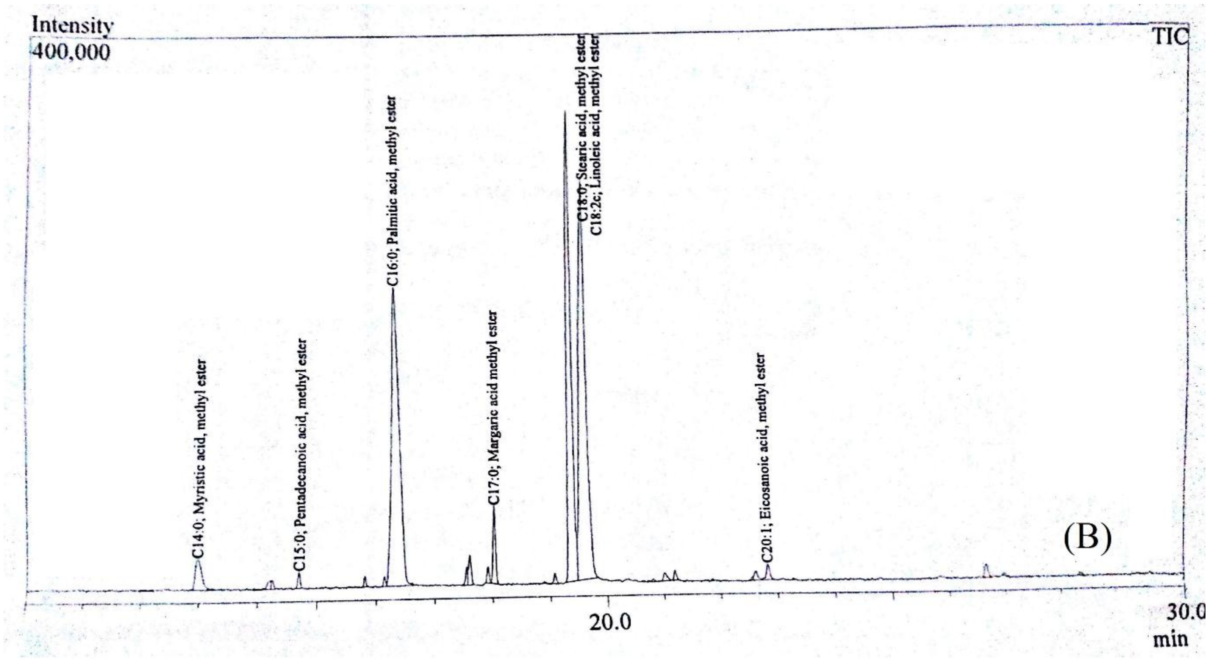

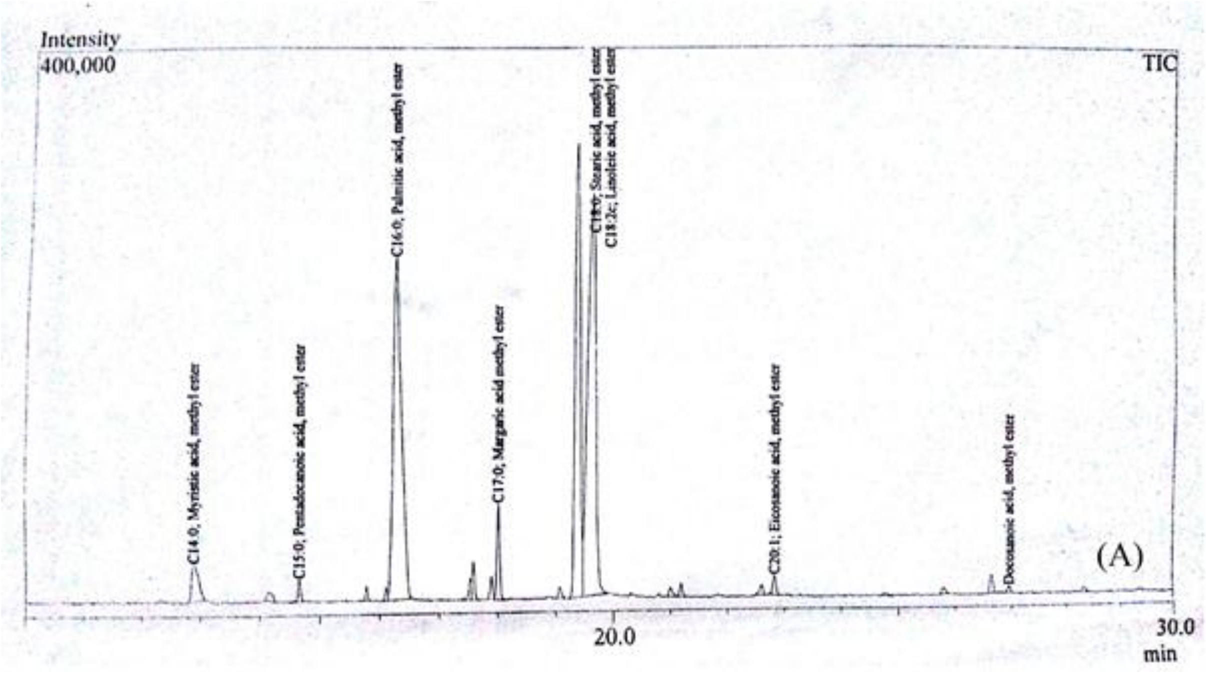

Supplementary Figure. Representative total ion chromatogram (TIC) of fatty acids using GC-MS of tallow (A) control tallow, and (B) oxidized tallow.
